# Treatment of Pediatric Displaced Intraarticular Olecranon Fractures with Resorbable Poly-L-Lactic-Co-Glycolic Acid (PLGA) Pins and Polydioxanone (PDS) Loops

**DOI:** 10.3390/children12030316

**Published:** 2025-02-28

**Authors:** Zoltán Tóth, Tamás Kassai, Marcell Varga, Tibor Molnár, Eszter Antal, Anna Gabriella Lamberti, Hermann Nudelman, Aba Lőrincz, Gergő Józsa

**Affiliations:** 1 Division of Surgery, Traumatology, Urology and Otorhinolaryngology, Department of Pediatrics, Clinical Complex, University of Pécs, 7 József Attila Street, 7623 Pécs, Hungary; toth.zoltan3@pte.hu (Z.T.); molnar.tibor97@gmail.com (T.M.); lamberti.anna@pte.hu (A.G.L.); dr.jozsa.gergo@gmail.com (G.J.); 2 Department of Pediatric Traumatology, Péterfy Hospital, Manninger Jenő National Trauma Center, 17 Fiumei Street, 1081 Budapest, Hungary; kassai.tamas@obsi.hu (T.K.); drvmarcell@gmail.com (M.V.); 3 Department of Thermophysiology, Institute for Translational Medicine, Medical School, University of Pécs, 12 Szigeti Street, 7624 Pécs, Hungary; zoltan.toth.dr@gmail.com (E.A.); nuhwaao.pte@tr.pte.hu (H.N.)

**Keywords:** pediatric, elbow, olecranon, fracture, resorbable, PLGA, PDS, implant

## Abstract

Background: Pediatric olecranon fractures, though rare, can be serious. Treatment varies by type and severity; displaced, intraarticular fractures usually need surgery, where biodegradable implants are emerging as an encouraging option to metal hardware. Methods: Our prospective, single-center, single-arm case series evaluates three pediatric olecranon fracture patients treated by resorbable poly-L-lactic-co-glycolic acid (PLGA) pins and polydioxanone (PDS) loops between Jan 2022 and January 2023 at the Department of Pediatrics, University of Pécs, Clinical Complex. Results: All patients achieved complete radiological healing with excellent functional recovery and no implant-related complications at one-year follow-up. Conclusions: Resorbable PLGA pins and PDS loops provide a promising alternative to conventional metallic fixation in pediatric olecranon fractures, abolishing the need for a second implant-removal surgery while maintaining stability and biocompatibility. Our findings support the child-friendly nature and growing role of biodegradable materials in pediatric fracture management.

## 1. Introduction

Olecranon, the proximal articular ulnar region, accounts for about 5% of all pediatric cubital fractures [1], and approximately 20% are complicated by other ipsilateral elbow injuries [2]. Primarily (68%) male patients are affected by these traumas. Mean ages ranged from 7.3 to 13.5 years, and the youngest documented patient was two months old, according to a recent systematic review [3]. High-energy direct blows or falling to an outstretched hand or a flexed elbow during sports activities are conventional etiological mechanisms [4].

Classification systems for pediatric olecranon fractures vary—with methods from Caterini [2], Horne and Tanzer [5], Mayo [6], to the Arbeitsgemeinschaft für Osteosynthesefragen (AO) Foundation developed Pediatric Comprehensive Classification of Fractures (PCCF) protocol [7]—yet no single approach has gained universal acceptance.

Initial diagnosis relies on elbow X-rays (anteroposterior (AP) and lateral views) [8] using specific alignment markers (anterior humeral– and radial head–capitellar lines, Baumann angle [9], along with the posterior fat pad sign for occult fractures [10]), with computer tomography (CT), magnetic resonance imaging (MRI), or ultrasound serving as adjuncts when further detail is needed.

Recognizing the predictable ossification of six elbow centers [11] via the CRITOE mnemonic (C: capitellum, R: radial head, I: internal (medial) epicondyle, T: trochlea, O: olecranon, E: external (lateral) epicondyle) [9] is key to distinguishing normal development from fractures, although contralateral imaging is generally discouraged due to individual variability [12].

Generally, stable, non-displaced fractures can be treated by a conservative method employing cast immobilization. Displaced intraarticular olecranon fractures are primarily treated with open reduction and internal fixation (ORIF), utilizing various fixation methods such as plate osteosynthesis (POS), compression screws, tension band wires (TBW), or tension band sutures (TBS) [4]. Special care must be taken to avoid damaging the growth plate, as this could lead to long-term functional deficits or growth disturbances.

Tension band fixation remains the standard treatment for displaced transverse intraarticular olecranon fractures, though concerns of increased fixation revision risk have arisen in older (>14 years) and heavier (>50 kg (kgs)) patients when using TBS compared to TBW [1,13]. Developed by Weber and Vasey in 1963 [14], and endorsed by the AO Foundation, the TBW technique involves reducing the fracture, inserting two parallel K-wires through the olecranon, and securing them with a twisted figure-of-eight steel wire passed through a transverse ulna hole [15]. Although some advocate placing this hole anterior to the intramedullary pin for enhanced rigidity [16], biomechanical studies have not confirmed a significant advantage [17]. Despite effectively compressing the fracture, TBW is often associated with painful hardware, leading to implant removal in up to 88% of cases, particularly in active children [4,18,19]. In certain hospitals, the K-wire tips are intentionally left protruding through the skin (i.e., proud or exposed) to ease later removal and reduce operative burden, though, it increases the chance of pin-site infection, which necessitates proper monitoring and hygiene [20]. Because K-wire and the cerclage ends were irritating in many cases, alongside K-wire fixation, a special absorbable suture (ultrahigh-molecular-weight braided polyester suture; FiberWire) was used to create the figure-of-eight TBS, which was found to be more advantageous in this regard [21].

Among surgical treatments, compression screw utilization has also gained attention, but in many cases, the screw tip above the olecranon causes discomfort for the patients [22]. However, Herbert screws also showed minor postoperative complications (implant loosening, bursitis) and better clinical outcomes (QuickDASH (Disabilities of the Arm, Shoulder, and Hand) score, intraoperative blood loss, and surgery duration) compared to TBW in adolescents by a recent study [23]. A previous adult study proved a new enhancement to the AO TBW technique by incorporating bioabsorbable magnesium (Mg) compression screws along with polyethylene mesh tape [24]. The latter approach provides the biomechanical advantages of TBW fixation while eliminating the concerns associated with hardware. Following reduction, POS fixation can also be an appropriate solution, provided that significant growth is no longer expected in the child [25].

In recent decades, biodegradable polyesters such as poly-glycolic acid (PGA) and poly-lactic acid (PLA) have been developed for medical use. An important benefit is their bending modulus, which is similar to that of bone, preventing complications associated with stress shielding, unlike when using metal hardware. Poly-L-lactic-co-glycolic acid (PLGA) polymer implants retain their mechanical strength and properties for at least eight weeks before undergoing full biodegradation into water and carbon dioxide (CO_2_) within approximately two years [26,27]. An optimal balance between the strength and degradation rate is provided by the specific 85L:15G composition (85% lactic acid and 15% glycolic acid) with excellent biocompatibility. Specific, composition-controlled absorption allows for continuous bone remodeling and the recanalization of the pin-formed place. However, temperature, pH, and the presence of enzymes, cell mediated-, or other catalysts may influence their degradation rate [28].

Our previous cohort study showed that pediatric medial humeral epicondyle fractures could be effectively managed by PLGA pins and absorbable sutures [29]. However, according to our literature search, currently, no study evaluated the clinical and radiological outcomes of resorbable PLGA pins and polydioxanone (PDS) loops in pediatric olecranon fractures. Therefore, this case series aims to evaluate these aforementioned bioresorbable alternatives to conventional metallic fixation.

## 2. Patients and Methods

### 2.1. Study Design and Patient Selection

Between January 2022 and January 2023, the Division of Surgery, Traumatology, Urology, and Otorhinolaryngology, Department of Pediatrics at the University of Pécs, Clinical Complex was involved in the consecutive treatment of three pediatric patients with olecranon fractures utilizing PLGA pin implants (Activa PIN™, Bioretec Ltd., Tampere, Finland) and PDS loops (PDS™ II, Ethicon, Johnson & Johnson, New Brunswick, NJ, USA). Our single-center, prospective case series aims to present adolescent olecranon fracture treatment with resorbable implants and evaluate the one-year follow-ups of the result of this method. Written informed consent was obtained from the guardians of the patients to participate in this study, which was approved by the Regional Research Ethics Committee, Clinical Complex, University of Pécs on 23 April 2021 (8737-PTE2021).

This case series involves three patients, which were included due to meeting the following criteria: (1) all under 18 years of age at the time of injury, with (2) open physes (growth plates), who (3) sustained displaced intraarticular olecranon fractures (4) requiring surgical intervention, (5) utilizing resorbable pin fixation and a PDS compression loop, and later (6) attended the one-year follow-up. During the investigation period, all patients were included who met the inclusion criteria.

### 2.2. Operative Algorithm

Under general anesthesia, the patient is placed in a supine position and an open surgical approach is performed to expose the fracture site. After achieving accurate reduction, temporary stabilization is obtained using K-wires. These wires not only hold the fracture in proper alignment but also serve to pre-drill pilot holes with a 2.0 mm K-wire—matching the diameter of the bioabsorbable pins intended for final fixation. Temporary K-wires are then replaced with PLGA implants, which are gently impacted rather than screwed—to ensure proper seating without compromising the implant’s integrity into the prepared holes so that a sufficient length remains protruding from the bone surface. Protruding segments are essential for the effective engagement of a PDS tension band, which is applied through a drilled canal distal to the fracture to generate the necessary compressive force. To secure the tension band, the excess length of the implants is addressed by cauterizing and trimming their ends with a specialized cautery tool; this process forms a shoulder on the pin that prevents the PDS loop from slipping off during tensioning. Exact protrusion distance is determined intraoperatively by the surgeon based on the specific anatomical requirements and desired tension band effect.

Postoperatively, the arm is immobilized in a dorsal above-elbow cast with the elbow flexed at 90 degrees (°) for approximately three weeks. Once the right-angle cast is removed, active physiotherapeutic rehabilitation is initiated to restore function and range of motion.

## 3. Results

### 3.1. Case 1

**Figure 1:**
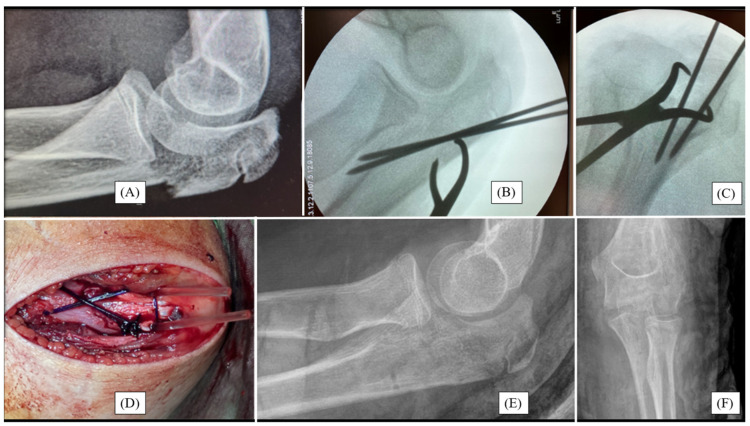
Primary X-ray of an olecranon fracture sustained by a 12-year-old boy, from lateral view (**A**). Fluoroscopic images after open reduction and temporary K-wire fixation (**B**,**C**). Intraoperative photograph showing the two parallel resorbable pins and a PDS compression loop (**D**). Postoperative X-rays demonstrate good alignment and perfect articular congruency from lateral (**E**) and AP (**F**) aspects.

While cycling, a 12-year-old boy injured his left elbow. Initial X-rays confirmed a Mayo type II/A olecranon fracture (Figure 1A). Due to the formation of joint steps, surgical intervention was necessary. Throughout the open approach, the fracture was reduced to an anatomical position and temporarily fixed with two K-wires (Figure 1B). After the removal of the K-wires, the absorbable PLGA pins were placed into the cavity previously created by the wires. Subsequently, compression was achieved by PDS loops placed in a drilled canal distal to the fracture to ensure proper stability (Figure 1D). A right-angle dorsal cast was employed to immobilize the left upper limb for three weeks, followed by active physiotherapy treatment.

**Figure 2:**
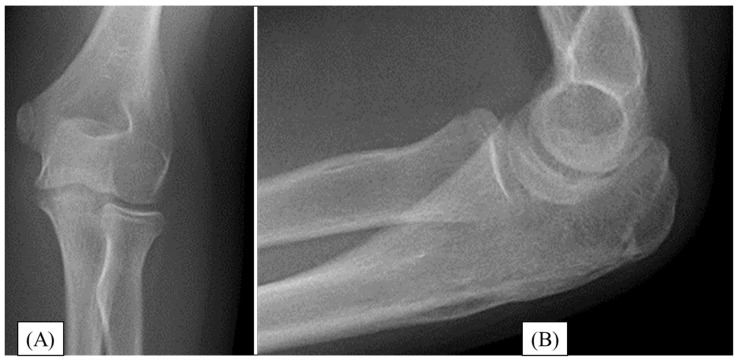
One year after the reconstruction of the olecranon fracture. Control X-rays show perfect articular congruency and a completely healed fracture from AP (**A**) and lateral views (**B**).

During follow-up, the child’s range of motion (ROM) in the elbow gradually improved with regular physiotherapy. Three months after the surgery, the elbow ROM was fully restored. At the one-year follow-up, the motion of the left elbow was identical to that of the unaffected side. No sensitivity or irritation was reported around the surgical site. Follow-up X-rays showed complete healing of the fracture site with full joint congruence (Figure 2). One-year postoperatively, the child was symptom-free and has returned to everyday sports activities.

### 3.2. Case 2

**Figure 3:**
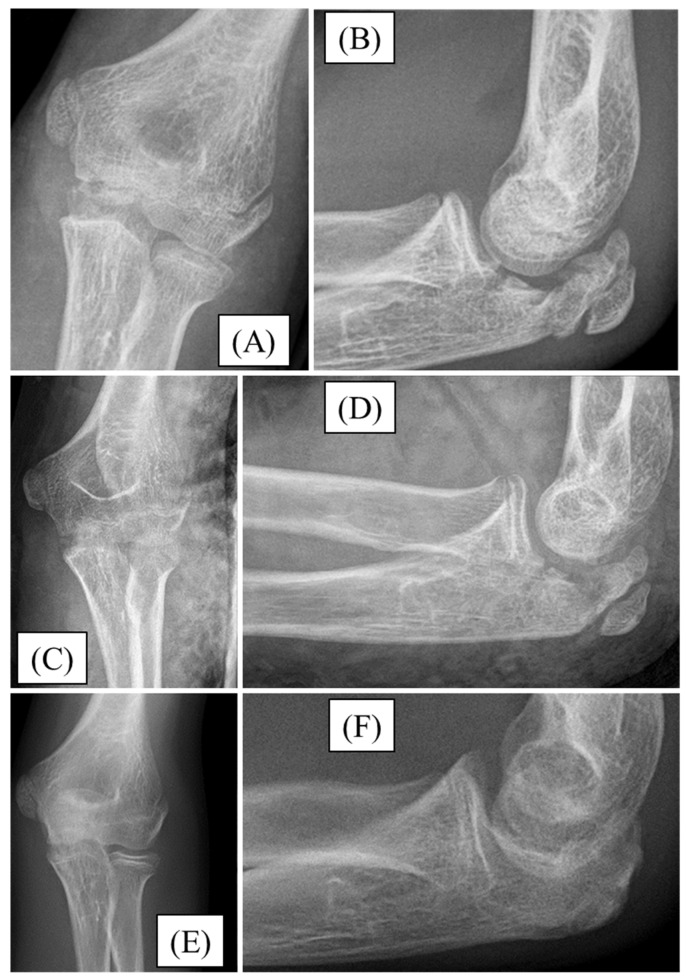
Preoperative X-rays from AP (**A**) and lateral (**B**) aspects of olecranon fracture. Postoperative X-rays show good reduction and perfect articular congruency; AP (**C**) and lateral (**D**). One year later, the fracture was entirely healed with no sign of articular incongruency (**E**,**F**).

After falling down the stairs, a 12-year-old boy sustained an injury. Significant swelling and restricted joint movement developed above his left elbow. A Mayo type II/B olecranon fracture with joint step formation was confirmed by X-rays (Figure 3A,B). After preparation, the fracture was reduced and stabilized with absorbable PLGA pins and a PDS loop. Follow-up radiographs showed the fracture in good alignment (Figure 3C,D) with an uneventful postoperative period. For three weeks, the left upper limb was restrained in a long arm cast. Following the restriction, the child received active physiotherapy. All through the follow-up, we observed the gradual mending of the fracture. One-year follow-up radiographs confirmed finished fracture healing, with complete elbow joint function (Figure 3E,F).

### 3.3. Case 3

**Figure 4:**
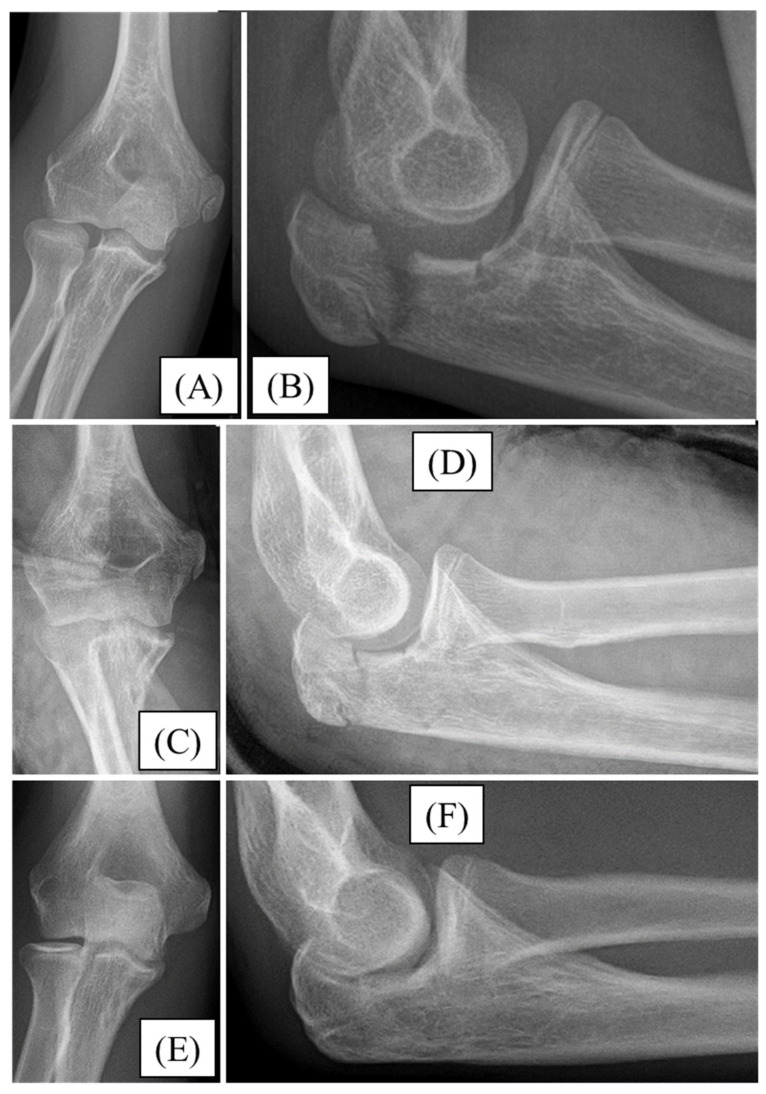
Initial X-rays of the right olecranon fracture (AP (**A**), lateral (**B**)), and the postoperative radiographs show adequate articular alignment (AP (**C**), lateral (**D**)). One year later the X-ray images (AP (**E**), lateral (**F**)) depict good alignment and acceptable congruency.

A 13-year-old boy injured his right elbow while cycling. He presented with significant swelling and restricted ROM. X-rays confirmed a comminuted Mayo type II/A olecranon fracture with joint involvement (Figure 4A,B). During open surgery, after achieving the most accurate reduction, the fracture was stabilized with absorbable implants (PLGA pins and a PDS loop) (Figure 4C,D), and the postoperative period proceeded without complications. After three weeks of casting, the child underwent active physiotherapy. One-year follow-up showed complete healing of the fracture on X-ray (Figure 4E,F), with full elbow ROM.

Summary patient and injury characteristics are collected in Table 1.

## 4. Discussion

Granting no far-reaching conclusions can be drawn from the results of these three instances, the presented cases focus on the potential of using a new method for treating pediatric and adolescent olecranon fractures in a more comfortable manner.

Pediatric olecranon fracture management must be approached with consideration for the growing skeletal system, as improper treatment can affect both function and future growth. Despite many advancements, the continued use of multiple classification systems underscores the lack of consensus on a standardized framework for assessing fracture severity and guiding optimal management in pediatric patients [4]. Treatment options for these injuries generally depend on the age of the child and the nature of the fracture [10,11]. Displaced or comminuted fractures often require surgical intervention. Here, the goal is to restore the anatomical alignment of the olecranon and allow for optimal functional recovery. Operative techniques typically include fixation with screws, TBW, TBS, or POS [1,4,14,19,22,25,30,31]. Due to the great surgical burden (35% of all surgeries may be hardware removals [32]), and complications (such as pin-site infection, soft-tissue irritation, and skin perforation caused by wire ends) observed with metal implants, an increasing number of studies have presented their results achieved with resorbable implants [1,4,19].

Our case report discussed the treatment of three children with displaced, intraarticular olecranon fractures using the new PLGA resorbable technique. The surgical procedure, similar to interventions with metal implants, involved accurate repositioning and ensuring articular surface congruence. Fractures were then stabilized using resorbable PLGA pins, and then a PDS loop was applied to achieve adequate compression. Consequently, this modified technique, which closely follows traditional AO methods while incorporating biodegradable implants, ensures both mechanical stability and effective tension band application. After surgery, the operated limb was restrained in a cast for three weeks, followed by active physiotherapy to facilitate the swiftest recovery of function. No complications were observed during the follow-up examinations. At the one-year follow-up, all three children showed complete functional and radiological healing of the fractures, with good joint congruence and minimal scarring.

Despite these promising results, critical points from biomechanical studies must be deliberated. Although PLGA has a similar elastic modulus (measured in gigapascals or GPa) and exhibits greater elongation than cortical bone, its density (g/cm^3^), compressive and tensile yield strengths (MPa), and fracture toughness (MPa·m^1/2^) are lower than those of bone, 316L stainless steel, titanium, and Mg [33,34,35]. However, the absorbable pin undergoes autocompression when it comes into contact with blood and under the effect of body temperature. Then, the implant minimally shortens, and its diameter slightly increases. A mechanical comparative clinical study using K-wires was conducted to compare childhood radial condyle injuries, and the results have been published with no significant difference observed in terms of surgical stability [36].

PLGA implants have been shown to cause less inflammation and irritation in the surrounding tissues [37]. In contrast, the previous generation of implants made from PGA faced challenges due to their rapid degradation. Their byproducts activated pro-inflammatory cytokines (mainly C5a complements), leading to significant inflammation and poor clinical outcomes [4,38]. Moreover, due to their porous nature, PLGA can be combined well with other materials, such as antibiotics or chemotherapeutic agents, and may be designed for shorter or even ultra-long, controlled drug delivery [39]. It may be further refined for specific use cases, e.g., when mixed with kartogenin to induce hyaline chondrogenesis [40]. Additive, 3D-printed manufacturing may be an option for personalized implant dimensions, combination, structure, porosity, and surface to drastically alter the aforementioned physical resilience parameters [41].

As a biodegradable alternative, Mg compression screws with polyethylene bands should be considered. Mg ions released during electrochemical corrosion can promote osteogenesis and are generally considered safe, but excessive release can lead to local alkalization and tissue irritation [42]. Hydrogen gas (H_2_) production can cause gas bubbles in tissues, potentially leading to inflammation or discomfort. Mg hydroxide (Mg(OH)_2_) initially forms a protective layer, which may be eroded early by chloride ions (Cl^−^), leading to expedited resorption. In adult coronoid fracture patients, it only showed a minor, non-functional extension deficit [24]. However, delayed healing, pain, swelling, screw fracture, and loosening, and extensive resorption cysts have been noted in other areas by recent systematic reviews [32,43].

Several limitations restrict the general applicability of this trial. Statistical tests could not be run, due to not meeting the participant limit criteria. Nevertheless, due to the obsoleteness of the second, metal removal surgeries, it can be stipulated that the benefits of resorbable implants, when employed in other regions, are similarly applicable to coronoid fractures. Clinically, advantages such as reduction in surgery-related complications (e.g., bleeding, infection, scarring, and nerve damage), average narcosis, ionizing radiation exposure, hospitalization duration, stress levels, along with the related total costs of the procedures were observed during the investigation period, but could not be statistically proven, due to the lack of a control group and low number of participants. As the trial was conducted in a single regional center, with only Caucasian and male participants, socio-economic, racial, and gender bias may be present. Therefore, a greater population, and at least single-blinded, randomized controlled trials (RCTs) are needed to further substantiate the qualities and constraints of resorbable implants for pediatric olecranon fractures.

## 5. Conclusions

The management of pediatric olecranon fractures requires careful consideration of both immediate fracture stabilization and long-term outcomes. Biodegradable PLGA implants and PDS loops represent a promising approach, offering the benefit of eliminating the need for removal surgery. It may be a significant advantage in a growing child by potentially reducing anesthetic and imaging radiation dosages, discomfort, hospitalization length, and financial burden related to an additional operation, the degree of which still requires extensive research. Furthermore, their use must be weighed against concerns related to strength, resorption rates, high initial costs, and surgical expertise required due to visualization difficulties. Ultimately, a personalized treatment plan that considers the individual needs of the child, the nature of the fracture, and the available resources will ensure the best outcomes for these patients.

## Figures and Tables

**Table 1:** Demographic data and olecranon fracture feature synopses of pediatric patients.

Patient	1	2	3
Age	12	12	13
Sex	boy	boy	boy
Side	left	left	right
Mechanism	cycling	stair fall	cycling
Mayo severity	II/A	II/B	II/A
Complications	no	no	no

## Data Availability

Data are contained within the article.

## References

[1] Perkins C.A., Busch M.T., Christino M.A., Axelrod J., Devito D.P., Fabregas J.A., Flanagan J.C., Murphy J., Olszewski D., Schmitz M.L. (2018). Olecranon Fractures in Children and Adolescents: Outcomes Based on Fracture Fixation. J. Child. Orthop..

[2] Caterini R., Farsetti P., D’Arrigo C., Ippolito E. (2002). Fractures of the Olecranon in Children. Long-Term Follow-up of 39 Cases. J. Pediatr. Orthop. Part B.

[3] Holme T.J., Karbowiak M., Arnander M., Gelfer Y. (2020). Paediatric Olecranon Fractures: A Systematic Review. EFORT Open Rev..

[4] De Maio F., Gorgolini G., Caterini A., Luciano C., Covino D., Farsetti P. (2022). Treatment of Olecranon Fractures in Childhood: A Systematic Review. Front. Pediatr..

[5] Horne J.G., Tanzer T.L. (1981). Olecranon Fractures: A Review of 100 Cases. J. Trauma.

[6] Morrey B.F. (1995). Current Concepts in the Treatment of Fractures of the Radial Head, the Olecranon, and the Coronoid. JBJS.

[7] Joeris A., Lutz N., Blumenthal A., Slongo T., Audigé L. (2017). The AO Pediatric Comprehensive Classification of Long Bone Fractures (PCCF). Acta Orthop..

[8] Iyer R.S., Thapa M.M., Khanna P.C., Chew F.S. (2012). Pediatric Bone Imaging: Imaging Elbow Trauma in Children???A Review of Acute and Chronic Injuries. Am. J. Roentgenol..

[9] DeFroda S.F., Hansen H., Gil J.A., Hawari A.H., Cruz A.I. (2017). Radiographic Evaluation of Common Pediatric Elbow Injuries. Orthop. Rev..

[10] Fujihara Y., Tatebe M., Fujihara N., Tanaka H., Hirata H. (2017). Useful Plain Radiographic Findings in Diagnosis of Pediatric Olecranon Fracture Complicated With Proximal Radial Fracture. Pediatr. Emerg. Care.

[11] Cheng J.C., Wing-Man K., Shen W.Y., Yurianto H., Xia G., Lau J.T., Cheung A.Y. (1998). A New Look at the Sequential Development of Elbow-Ossification Centers in Children. J. Pediatr. Orthop..

[12] Khong P.-L., Ringertz H., Donoghue V., Frush D., Rehani M., Appelgate K., Sanchez R., ICRP (2013). *ICRP Publication* 121: Radiological Protection in Paediatric Diagnostic and Interventional Radiology. Ann. ICRP.

[13] Chalidis B.E., Sachinis N.C., Samoladas E.P., Dimitriou C.G., Pournaras J.D. (2008). Is Tension Band Wiring Technique the “Gold Standard” for the Treatment of Olecranon Fractures? A Long Term Functional Outcome Study. J. Orthop. Surg..

[14] Müller M.E., Allgöwer M., Schneider R., Willenegger H. (2013). Manual der Osteosynthese: AO-Technik.

[15] Weber B.G., Vasey H. (1963). Osteosynthesis in olecranon fractures. Z. Unfallmedizin Berufskrankh. Rev. Med. Accid. Mal. Prof..

[16] Rowland S.A., Burkhart S.S. (1992). Tension Band Wiring of Olecranon Fractures. A Modification of the AO Technique. Clin. Orthop..

[17] Paremain G.P., Novak V.P., Jinnah R.H., Belkoff S.M. (1997). Biomechanical Evaluation of Tension Band Placement for the Repair of Olecranon Fractures. Clin. Orthop..

[18] Gaddy B.C., Strecker W.B., Schoenecker P.L. (1997). Surgical Treatment of Displaced Olecranon Fractures in Children. J. Pediatr. Orthop..

[19] Romero J.M., Miran A., Jensen C.H. (2000). Complications and Re-Operation Rate after Tension-Band Wiring of Olecranon Fractures. J. Orthop. Sci..

[20] Suganuma S., Tada K., Takagawa S., Yasutake H., Shimanuki K., Shinmura K., Fujita K., Tsuchiya H. (2022). Comparing Exposed and Buried Kirschner Wires in Fixation for Pediatric Supracondylar Humerus Fractures: A Propensity Score-Matched Study. J. Orthop. Surg. Hong Kong.

[21] Gortzak Y., Mercado E., Atar D., Weisel Y. (2006). Pediatric Olecranon Fractures: Open Reduction and Internal Fixation with Removable Kirschner Wires and Absorbable Sutures. J. Pediatr. Orthop..

[22] Mun F., Suresh K.V., Hayashi B., Margalit A., Sponseller P.D., Lee R.J. (2023). Compression Screw Fixation for Pediatric Olecranon Fractures. J. Pediatr. Orthop..

[23] Yang W., Zhang X., Sun D., Jin S., Chen J., Li Y. (2024). Outcomes of Olecranon Fractures in Adolescents: Comparison of Tension Band Wiring and Herbert Screw Fixations. Front. Pediatr..

[24] Crozier-Shaw G., Mahon J., Bayer T.C. (2020). The Use of Bioabsorbable Compression Screws & Polyethylene Tension Band for Fixation of Displaced Olecranon Fractures. J. Orthop..

[25] Inui A., Kuroda T., Kurosawa T., Kokubu T., Mifune Y., Nishimoto H., Kuroda R. (2018). Case Series of Comminuted Olecranon Fracture Treated by Plate Fixation; Do We Have to Remove the Plate?. Kobe J. Med. Sci..

[26] Eppley B.L., Reilly M. (1997). Degradation Characteristics of PLLA-PGA Bone Fixation Devices. J. Craniofac. Surg..

[27] Perhomaa M., Pokka T., Korhonen L., Kyrö A., Niinimäki J., Serlo W., Sinikumpu J.-J. (2021). Randomized Controlled Trial of the Clinical Recovery and Biodegradation of Polylactide-Co-Glycolide Implants Used in the Intramedullary Nailing of Children’s Forearm Shaft Fractures with at Least Four Years of Follow-Up. J. Clin. Med..

[28] van Apeldoorn A.A., van Manen H.-J., Bezemer J.M., de Bruijn J.D., van Blitterswijk C.A., Otto C. (2004). Raman Imaging of PLGA Microsphere Degradation inside Macrophages. J. Am. Chem. Soc..

[29] Kassai T., Varga M., Józsa G. (2022). Pediatric Medial Humeral Epicondyle Fracture in Children: Are Biodegradable Pins with Tension Band Absorbable Sutures Efficient?. Medicine.

[30] Matthews J.G. (1980). Fractures of the Olecranon in Children. Injury.

[31] Newell R.L. (1975). Olecranon Fractures in Children. Injury.

[32] Baldini M., Coppa V., Falcioni D., Senigagliesi E., Marinelli M., Gigante A.P. (2021). Use of Resorbable Magnesium Screws in Children: Systematic Review of the Literature and Short-Term Follow-up from Our Series. J. Child. Orthop..

[33] Gay J.F., King L.S., Lysen A.T. (2014). Use of Magnesium Alloys in Tension Band Wiring of Olecranon Fractures.

[34] Melo L., Salmoria G., Fancello E., Roesler C. (2017). Influence of Processing Conditions on the Mechanical Behavior and Morphology of Injection Molded Poly (Lactic-Co-Glycolic Acid) 85:15. Int. J. Biomater..

[35] Losertova M., Štamborská M., Lapin J., Mareš V. (2016). *Comparison of Deformation Behavior of* 316L Stainless Steel and Ti6Al4V Alloy Applied in Traumatology. Metal.-Sisak Then Zagreb..

[36] Kassai T., Krupa Z., Józsa G., Hanna D., Varga M. (2024). Comparison of Biodegradable and Metallic Tension-Band Fixation for Paediatric Lateral Condyle Fracture of the Elbow. Injury.

[37] Ma S., Feng X., Liu F., Wang B., Zhang H., Niu X. (2021). The Pro-Inflammatory Response of Macrophages Regulated by Acid Degradation Products of Poly(Lactide-Co-Glycolide) Nanoparticles. Eng. Life Sci..

[38] Ceonzo K., Gaynor A., Shaffer L., Kojima K., Vacanti C.A., Stahl G.L. (2006). Polyglycolic Acid Induced Inflammation. Tissue Eng..

[39] Benhabbour S.R., Kovarova M., Jones C., Copeland D.J., Shrivastava R., Swanson M.D., Sykes C., Ho P.T., Cottrell M.L., Sridharan A. (2019). Ultra-Long-Acting Tunable Biodegradable and Removable Controlled Release Implants for Drug Delivery. Nat. Commun..

[40] Warren J.D. (2021). Bio-Printed, Bio-Functionalized PLGA-KGN Scaffolds as an Augmentation to Microfracture. Honor’s Thesis.

[41] Gupta A.K., Choudhari A., Kumar A., Gupta A. (2024). Custom Implants and Beyond: The Biomedical Potential of Additive Manufacturing. Arch. Case Rep..

[42] Pogorielov M., Husak E., Solodivnik A., Zhdanov S. (2017). Magnesium-Based Biodegradable Alloys: Degradation, Application, and Alloying Elements. Interv. Med. Appl. Sci..

[43] Antoniac I., Miculescu M., Mănescu (Păltânea) V., Stere A., Quan P.H., Păltânea G., Robu A., Earar K. (2022). Magnesium-Based Alloys Used in Orthopedic Surgery. Materials.

